# Prevalence of Persistent Left Superior Vena Cava in Patients with Congenital Heart Disease: A Systematic Review with Meta-Analysis

**DOI:** 10.3390/life16081281

**Published:** 2026-08-03

**Authors:** George Triantafyllou, Ioannis Paschopoulos, Daniel Gondorf, Niki Lama, Evangelos Oikonomou, Juan Jose Valenzuela-Fuenzalida, Juan Sanchis-Gimeno, Jose E. León-Rojas, Charalambos Vlachopoulos, Gerasimos Siasos, Maria Piagkou

**Affiliations:** 1 Department of Anatomy, School of Medicine, Faculty of Health Sciences, National and Kapodistrian University of Athens, 11527 Athens, Greece; georgerose406@gmail.com (G.T.); johnpascho@gmail.com (I.P.); daniel.gondorf@gmail.com (D.G.); mapian@med.uoa.gr (M.P.); 2 Research Unit of Radiology and Medical Imaging, School of Medicine, Faculty of Health Sciences, National and Kapodistrian University of Athens, 11528 Athens, Greece; niklampatr@gmail.com; 3 Second Department of Radiology, General University Hospital “Attikon”, National and Kapodistrian University of Athens, 12462 Athens, Greece; 4 Third Department of Cardiology, Sotiria Chest Disease Hospital, School of Medicine, Faculty of Health Sciences, National and Kapodistrian University of Athens, 11527 Athens, Greece; boikono@gmail.com (E.O.); gsiasos@med.uoa.gr (G.S.); 5 Faculty of Medicine, Andrés Bello University, Santiago 8370146, Chile; juan.kine.2015@gmail.com; 6 Department of Chemical and Biological Sciences, Faculty of Health Sciences, Bernardo O’Higgins University, Santiago 8370993, Chile; 7 GIAVAL Research Group, Department of Anatomy and Human Embryology, Faculty of Medicine, University of Valencia, 46001 Valencia, Spain; juan.sanchis@uv.es; 8 Grupo de Investigación Bienestar, Salud y Sociedad, Escuela de Psicología y Educación, Universidad de Las Américas, Quito 170124, Ecuador; 9 First Department of Cardiology, General Hospital of Athens “Hippokrateion”, School of Medicine, Faculty of Health Sciences, National and Kapodistrian University of Athens, 11527 Athens, Greece; cvlachop@otenet.gr

**Keywords:** persistent left superior vena cava, congenital heart diseases, cardiology, evidence-based anatomy, meta-analysis

## Abstract

Persistent left superior vena cava (PLSVC) is the most frequently reported anatomical variant of the thoracic systemic venous system. While its association with congenital heart disease (CHD) is well recognized, its prevalence within CHD populations and its relative occurrence compared with non-CHD cohorts remain incompletely characterized. This study aimed to estimate the pooled prevalence of PLSVC among patients with CHD and to assess its relative frequency compared with that in control populations. A systematic review of PubMed, Scopus, and Web of Science was conducted from database inception to January 2026. Studies reporting the prevalence of PLSVC in CHD cohorts were included. A random-effects meta-analysis was performed, and risk ratios (RRs) were calculated for studies that included both CHD and non-CHD groups. Sixteen studies (*n* = 37,370) were included. The pooled prevalence of PLSVC in CHD patients was 6.36% (95% CI: 4.70–8.24). Significant heterogeneity was identified (I^2^ = 97.6%). An increased likelihood of PLSVC was observed in CHD populations (RR = 15.34; 95% CI: 5.72–41.14). However, this relative risk is based on a highly limited number of comparative studies (*n* = 4) and exhibits substantial heterogeneity (I^2^ = 92.47%); thus, it must be interpreted with significant caution. Subgroup analyses did not demonstrate statistically significant differences based on nationality (*p* = 0.59), study type (*p* = 0.32) and age group (*p* = 0.95), while imaging modality was statistically significant (*p* = 0.01). PLSVC is observed in a notable proportion of patients with CHD, although variability in reported prevalence is substantial and likely influenced by methodological differences across studies. Given its potential implications for catheter-based and surgical procedures, systematic evaluation of thoracic venous anatomy should be considered during the diagnostic and preoperative assessment of patients with CHD.

## 1. Introduction

Congenital heart disease (CHD) represents the most frequent cause of major birth defects worldwide, affecting millions of newborns every year. Current estimates suggest a global birth prevalence ranging from 8.2 to 9.4 per 1000 live births [1,2]. Accurate evaluation of cardiac and vascular anatomy in these patients is pivotal, as many individuals require complex, multi-staged surgical or interventional procedures throughout their lives [3].

Persistent left superior vena cava (PLSVC) is the most frequently encountered developmental variant of the thoracic systemic venous system and arises from failure of regression of the left anterior cardinal vein during embryological development [4]. In most instances, the PLSVC drains into the right atrium via a dilated coronary sinus, which is often the first clinical indicator leading to the suspicion of this anomaly during imaging [4]. While the estimated prevalence of PLSVC in the general population is 0.61% [5], a significantly higher frequency is observed among patients with CHD [4].

The presence of PLSVC carries important clinical and procedural implications, particularly in patients undergoing invasive cardiovascular interventions [6]. Altered venous anatomy may complicate central venous access, right heart catheterization, and cardiac device implantation, often necessitating modified techniques and potentially increasing procedural complexity, duration, and radiation exposure [6].

Although the prevalence was established in the general population with our previous meta-analysis [5], the reported prevalence of PLSVC across the spectrum of CHD remains heterogeneous and fragmented. Therefore, the aim of this systematic review and meta-analysis is to establish the pooled prevalence of PLSVC in patients with CHD and determine the relative risk compared to the healthy population.

## 2. Materials and Methods

### 2.1. Methodology

This systematic review with meta-analysis was performed in adherence with the PRISMA 2020 [7] and Evidence-based Anatomy Workgroup [8] guidelines. To detect possible risk of bias for the included studies, the Anatomical Quality Assurance Tool (AQUA) was used [9]. The AQUA tool evaluates five domains—(1) objectives and subject characteristics, (2) study design, (3) methodology characterization, (4) descriptive anatomy, and (5) reporting of results—using standardized responses (“Yes”, “No”, or “Unclear”), which collectively inform an overall risk-of-bias classification (“Low”, “High”, or “Unclear”) [9]. This review was not registered in PROSPERO due to the temporary suspension of registrations for prevalence meta-analyses.

### 2.2. Search Analysis

Two independent reviewers (I.P. and D.G.) performed the literature search and data extraction. Any discrepancies were resolved through discussion and consensus with senior authors (G.T. and M.P.). The keywords “persistent left superior vena cava”, “congenital heart diseases”, “prevalence”, “incidence” and “study” were used in the online databases MEDLINE (PubMed), Scopus and Web of Science, until 31 January 2026. No database filters were applied to maximize sensitivity. The complete search strings for each database are provided in Table 1.

Studies reporting the PLSVC prevalence in patients with CHDs and/or studies comparing the prevalence of PLSVC between healthy and CHD patients were selected as eligible to answer our research question. Studies involving only a healthy population, case reports, animal studies, conference abstract and letters to the editor were excluded, as well as studies with irrelevant, insufficient or incomplete data. No language or data restrictions were imposed. Moreover, a hand-on search of the significant anatomical journals (Clinical Anatomy, Annals of Anatomy, Journal of Anatomy, Anatomical Record, Surgical and Radiological Anatomy, Folia Morphology, Anatomical Science International, Anatomy and Cell Biology, Morphologie and European Journal of Anatomy) and the references of all included studies was also performed. The data were extracted to Microsoft Excel sheets before statistical analysis. The following parameters were reported: authors, year of publication, nationality, demographics, modality used to identify the variant, sample of CHD patients, prevalence of PLSVC in CHD patients, sample of healthy (non-CHD) patients, and prevalence of PLSVC in healthy patients.

### 2.3. Statistical Analysis

Statistical analysis was conducted using the open-source R programming language and the RStudio software version 4.3.3 (RStudio Team, Boston, MA, USA) using the “meta” and “metafor” packages by a single researcher (G.T.). According to the guidelines for anatomical meta-analysis [8], the pooled prevalence was calculated with random-effects models and by using the inverse variance. As the current meta-analysis investigated the prevalence of PLSVC, it corresponded to a proportions meta-analysis that was conducted using the Freeman–Tukey double arcsine transformation, the DerSimonian–Laird estimator for the between-study variance tau^2^, and the Jackson method for confidence interval of tau^2^ and tau. For studies providing data on both CHD and healthy control populations, we calculated the risk ratio (RR). A random-effects model was employed to determine the relative likelihood of PLSVC in the CHD group compared to the healthy group. An RR > 1.0 with a *p*-value < 0.05 was considered statistically significant. Cochran’s Q statistic was calculated to evaluate the presence of heterogeneity across studies, and the Higgins I^2^ statistic quantified the heterogeneity. A Cochran’s Q *p*-value < 0.10 was considered significant. Higgins I^2^ values between 0 and 40% were regarded as might not necessary, 30–60% were regarded as moderate heterogeneity, 50–90% were characterized as substantial heterogeneity, and 75–100% may represent considerable heterogeneity [8]. Subgroup analysis for the impact of nationality and study type was done, as well as meta-regression analysis for the impact of the year of publication. For all the tests of the current meta-analysis, a *p*-value less than 0.05 was considered statistically significant. To evaluate the robustness of the pooled prevalence estimates and identify potential sources of heterogeneity, a leave-one-out sensitivity analysis was performed. This procedure involved systematically omitting one study at a time and recalculating the pooled effect size and the Higgins I^2^ statistic to determine if any single dataset disproportionately influenced the overall results. Moreover, a Baujat diagnostic plot was generated to visually identify studies that contributed disproportionately to the overall heterogeneity and those that exerted the greatest influence on the pooled effect size. To evaluate the presence of small-study effect (the phenomenon that smaller studies may show different effects than large ones), the DOI plot with LFK index was generated [10,11].

## 3. Results

### 3.1. Study Identification

**Figure 1:**
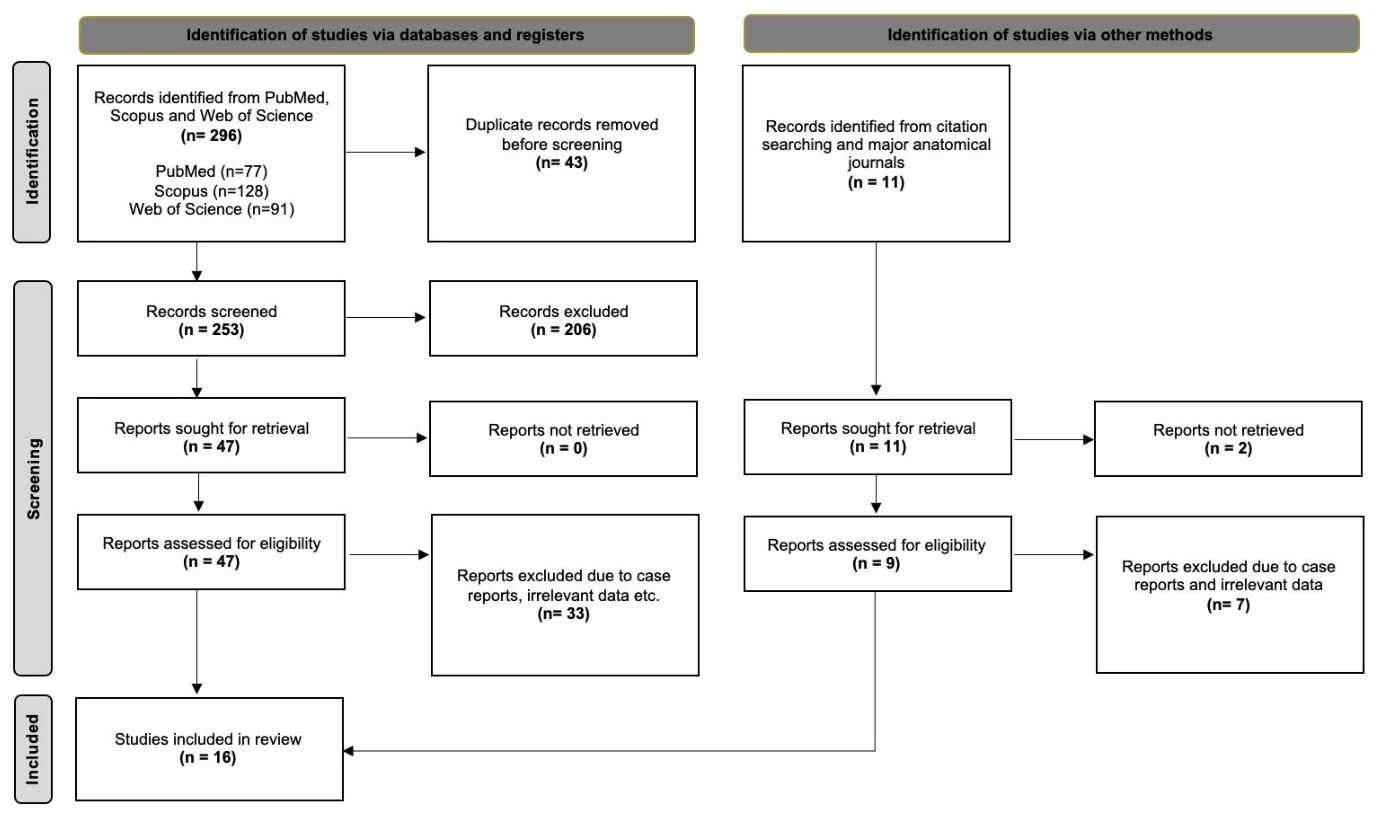
PRISMA 2020 flow chart for the systematic review analysis.

The initial database search yielded 296 records, which were exported to Mendeley (version 2.14.0; Elsevier, London, UK). After removal of duplicates and clearly irrelevant studies, 47 articles underwent full-text assessment for eligibility. In addition, two further studies were identified through manual screening of reference lists and targeted searches of major anatomical journals. Ultimately, 16 studies met the inclusion criteria and were incorporated into the present systematic review and meta-analysis. The study selection process is illustrated in the PRISMA 2020 flow diagram (Figure 1) [7].

### 3.2. Characteristics of the Included Studies

Sixteen (16) studies were included, with a sample of 37,370 patients. The mean sample per article was 2336 patients. The publication year ranged from 1980 to 2024. Ten (10) studies were based on imaging modalities (ultrasound, interventional radiology and computed-tomography angiography), four (4) studies were based on intraoperative observations (surgical), and two (2) studies were cadaveric (autopsy). Ten (10) studies were performed on an Asian population, four (4) studies on a European population and two (2) studies on an American population. According to the AQUA tool [9], 11 studies were considered “Low” risk of bias, and five studies were “High” risk of bias (Table 2).

### 3.3. Meta-Analysis Outcomes

**Figure 2:**
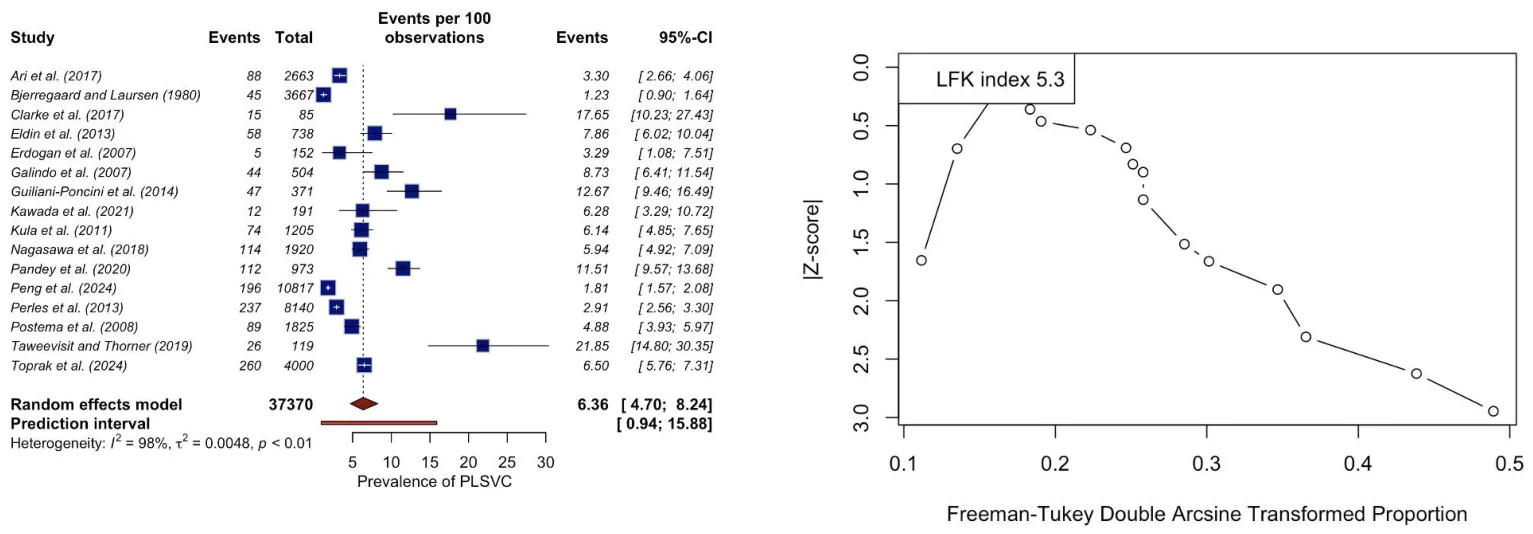
Forest and DOI plots for the estimated pooled prevalence of persistent left superior vena cava (PLSVC) in congenital heart disease patients [12,13,14,15,16,17,18,19,20,21,22,23,24,25,26,27].

Between the 16 studies (*n* = 37,370), the estimated PLSVC pooled prevalence was 6.36% (95% CI: 4.70–8.24) in CHD patients (Figure 2). The I^2^ test was calculated as 97.6%, indicating considerable heterogeneity. The leave-one-out analysis did not depict any outlier study influencing significantly the pooled prevalence (range: 6.03–7.28%) and the heterogeneity (range: 97.1–97.9%) (Supplementary Materials). The DOI plot had an LFK index of +5.3, indicating major asymmetry and possible small-study effect (Figure 2).

**Figure 3:**
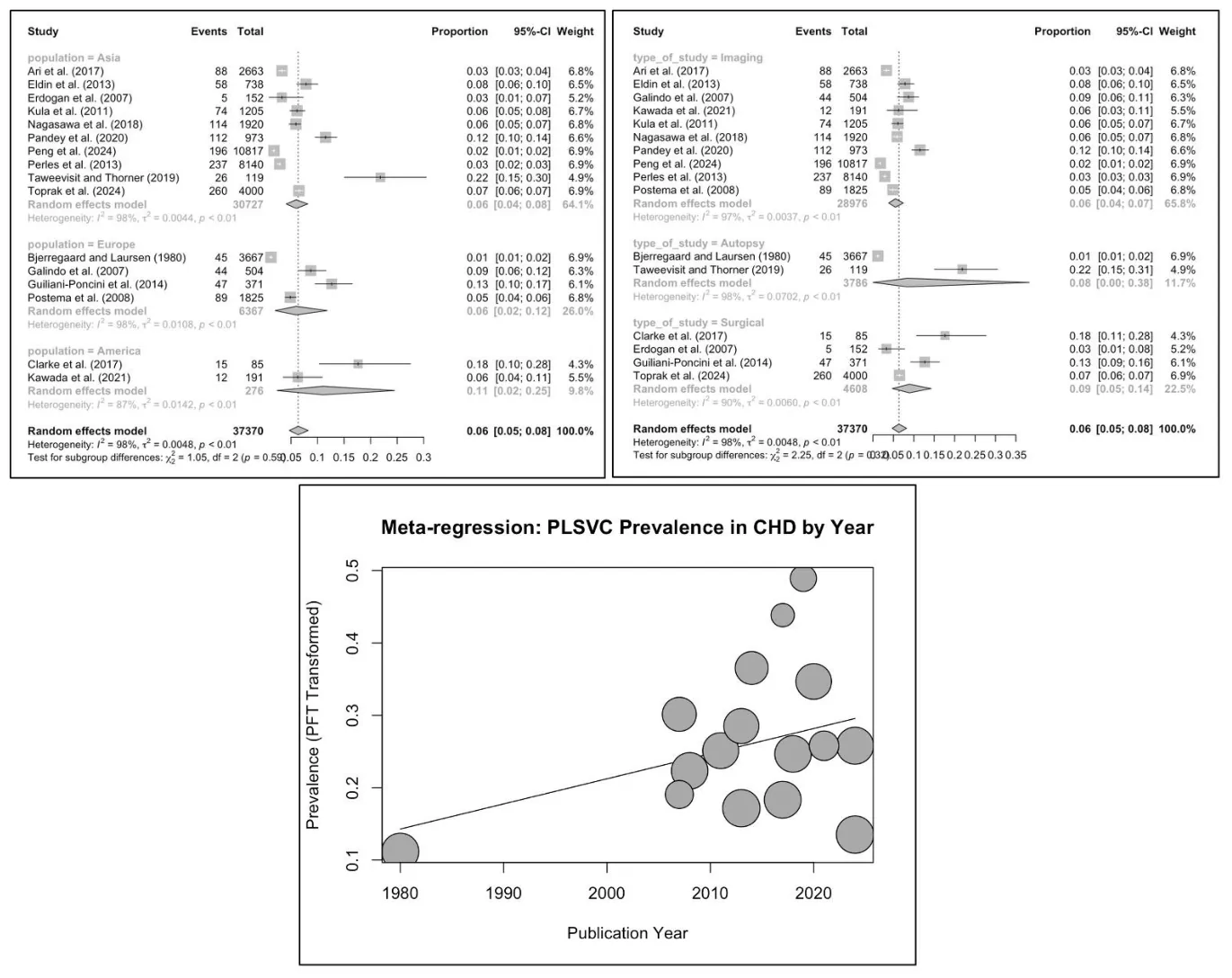
Subgroup analysis forest plot and meta-regression bubble plot for the estimated pooled prevalence of persistent left superior vena cava (PLSVC) in congenital heart disease patients, according to nationality, study type and year of publication [12,13,14,15,16,17,18,19,20,21,22,23,24,25,26,27].

The subgroup analysis according to nationality and study type did not depict statistically significant differences (*p* = 0.5904 and *p* = 0.3241, respectively) (Figure 3). A meta-regression analysis according to the publication year depicted an increasing tendency, but it was not statistically significant (*p* = 0.0592) (Figure 3).

The subgroup analysis between the prevalence of PLSVC according to the age group (*p* = 0.9456) and CHD type was not statistically significant (*p* = 0.1838) (Supplementary Materials) (Table 3). Stratification by specific imaging modality demonstrated a significant difference between echocardiography and CT (*p* = 0.0092); however, this result should be interpreted cautiously due to the low number of studies per group.

**Figure 4:**
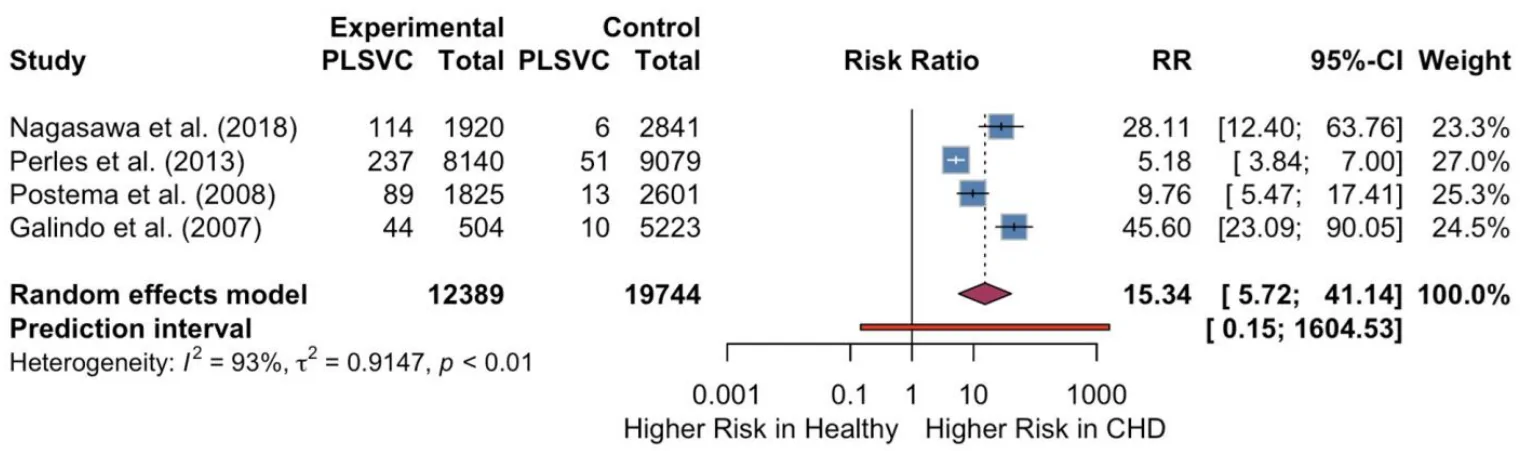
Forest plot for the risk ratio of the estimated pooled prevalence of persistent left superior vena cava (PLSVC) in congenital heart disease patients compared to healthy participants [17,21,24,25].

Four studies [n_1_ (control-healthy) = 19,744/n_2_ (experimental-CHD) = 12,389] reported the prevalence of PLSVC for both CHD and healthy patients. The RR was estimated as 15.34 (95% CI: 5.72–41.14). The I^2^ test was calculated as 92.47%, indicating considerable heterogeneity (Figure 4).

## 4. Discussion

The present systematic review and meta-analysis provide a comprehensive quantification of the prevalence of PLSVC in patients with CHD. Our results demonstrate a pooled prevalence of 6.36% among CHD patients, which represents a more than 15-fold increase compared to the healthy population. These findings confirm that, while PLSVC is a rare anatomical variant in the general population [5], it is significantly more frequent and a clinically significant finding in the context of congenital cardiac malformations.

During early embryological development, the thoracic venous system originates from two pairs of primary veins: the superior cardinal veins, which drain the cranial portion of the embryo, and the inferior cardinal veins, which return blood from the caudal region [28]. By the fifth week of gestation, these primitive vessels merge to form the bilateral common cardinal veins, which subsequently empty into the two horns of the sinus venosus [28]. This venous development occurs concurrently with early cardiac morphogenesis, wherein cardiogenic mesoderm cells converge to form the primitive straight tube heart within the pericardial cavity [29]. As development progresses into the eighth week, significant structural remodeling takes place to establish the definitive systemic venous circulation [28]. The superior transverse venous plexus develops into the normal left brachiocephalic vein, while the caudal portions of the right anterior and right common cardinal veins mature into the normal right-sided superior vena cava [28]. Concurrently, the developing heart tube undergoes crucial morphological changes, including rightward bulboventricular looping, convergence, and the initiation of cardiac septation, driven heavily by the progressive addition of progenitor cells from the second heart field [29,30]. In a normal embryological sequence, the caudal portion of the left superior cardinal vein and the left common cardinal vein regress and obliterate. Following this involution, they are integrated into the left-sided structures as a fibrous remnant known as the ligament of Marshall [28]. However, when the left common cardinal vein and the left anterior cardinal vein fail to undergo this programmed regression, they persist within the developing venous system as a PLSVC [28]. Consequently, the left horn of the sinus venosus, which typically forms the oblique vein and coronary sinus of the left atrium, receives this continuous anomalous venous return [28]. This altered embryological pathway explains why a PLSVC typically drains into the right atrium via a structurally dilated coronary sinus [28]. The intricate embryogenesis of the thoracic venous system overlaps temporally and spatially with critical stages of intracardiac morphogenesis, providing a developmental basis for the high prevalence of PLSVC in CHD cohorts. The primitive heart relies on a complex, highly regulated sequence of differential cell migration, looping, and endocardial cushion development to form the definitive four-chambered heart and its associated great vessels [29,30]. Any perturbation in these morphogenetic processes can exert widespread structural effects [29]. For instance, the abnormal widening of the atrioventricular canal, the alignment of the primitive ventricular septum, and the septation of the conotruncus all occur during the same specific window of the fifth to eighth weeks of gestation [29,30]. Hemodynamic alterations, early genetic perturbations, or primary developmental errors during this critical period not only disrupt intracardiac septation and looping—leading to anomalies such as atrioventricular septal defects, tetralogy of Fallot, or abnormal ventricular connections—but also simultaneously impede the normal obliteration of the left cardinal venous system [29]. Therefore, the persistence of the left superior vena cava is often not an isolated finding but rather a reflection of a broader developmental arrest that impacts the integrated morphogenetic pathways of the embryonic heart [29].

From an embryological and clinical anatomical perspective, the high-risk ratio (RR = 15.34) supports an association between abnormalities of cardiac morphogenesis and altered systemic venous development. PLSVC most commonly results from the failure of the left anterior cardinal vein to obliterate [6]. Our analysis suggests that this embryological ‘failure to regress’ is strongly associated with specific cardiac lesions. While our subgroup analysis did not show a statistically significant difference between CHD types, individual studies have highlighted strong associations with ventricular septal defects, double outlet right ventricle, and conotruncal anomalies [12,22]. Furthermore, the association with left-sided obstructive lesions, such as coarctation of the aorta, has been noted in prenatal settings [4], suggesting that altered hemodynamics in utero may play a role in the persistence of the left-sided caval vein. Rather than implying a direct causal relationship, this persistence likely reflects a shared developmental pathway with specific congenital cardiac anomalies.

The diagnostic modality used to identify PLSVC appears to substantially influence reported prevalence rates. Kula et al. (2011) demonstrated that transthoracic echocardiography detected PLSVC in only 2.6% of patients, whereas invasive angiography in the same cohort identified it in 6.1% [20]. This suggests that echocardiography—the primary screening tool for CHD—may significantly underreport this variant unless a dilated coronary sinus is specifically noted and further investigated with advanced imaging [20]. In contrast, multidetector CT angiography provides more comprehensive visualization of the thoracic venous system and is therefore particularly valuable for detailed anatomical assessment and preoperative planning [22]. Importantly, these differences should be interpreted in the context of methodological variability rather than true biological divergence, as detection of PLSVC is strongly influenced by imaging sensitivity, operator experience, and anatomical complexity.

The surgical and procedural implications of these findings are profound. In the context of CHD surgery, particularly for patients undergoing bidirectional cavopulmonary connections or Fontan procedures, the presence of a PLSVC necessitates specific modifications to the surgical plan to ensure adequate systemic venous drainage [3,27]. Toprak et al. (2025) [27] recently noted that, while PLSVC may not always increase mortality, it can complicate the intraoperative course and impact bypass times. Beyond surgery, PLSVC poses technical challenges for the placement of central venous catheters, pacemakers, and defibrillator leads, often leading to electrode displacement or unsuccessful cannulation [6].

Despite the strength of our pooled estimates, this meta-analysis revealed considerable heterogeneity. This heterogeneity is likely multifactorial and reflects differences in study design, diagnostic modality, patient selection, and temporal variation in clinical practice rather than random statistical fluctuation. The persistence of high heterogeneity following leave-one-out sensitivity analysis suggests that this variability is structural and inherent to the available literature. The LFK index of +5.3 indicates a small-study effect, suggesting that smaller studies with higher observed prevalences may be more likely to be published. Lastly, the subgroup analysis did not reach the minimum of three studies per group to be considered statistically reliable. Because of this extreme variability (depicted through heterogeneity and LFK index), the pooled prevalence estimates should be viewed as an approximation rather than a definitive metric. Similarly, while the calculated RR demonstrates a strong association between CHD and PLSVC, it is derived from only four comparative studies. This small sample size and high associated heterogeneity warrant cautious interpretation of the exact magnitude of the results.

## 5. Conclusions

Our meta-analysis establishes that the PLSVC is present in 6.36% of patients with CHD, while the relative risk compared to the healthy population is 15.34. This finding should be interpreted with caution due to the limited number of comparative studies and the considerable heterogeneity across the included data. A careful imaging assessment is required because a statistically significant difference between modalities was reported. These results advocate for the routine and systematic evaluation of the systemic venous anatomy in all CHD patients. A multidisciplinary approach involving cardiologists, radiologists, and surgeons is essential to mitigate the risks associated with invasive procedures and optimize surgical outcomes in this complex patient population.

## Figures and Tables

**Table 1:** Search combinations used in the current systematic review.

Database	Search Term Combinations
MEDLINE	((persistent left superior vena cava [Title/Abstract]) AND (congenital heart disease [Title/Abstract])) AND ((prevalence [Title/Abstract]) OR (incidence [Title/Abstract])) AND (study [Title/Abstract])
Scopus	TITLE-ABS-KEY ((“persistent left superior vena cava” OR “left superior vena cava”) AND (“congenital heart disease”) AND (prevalence OR incidence OR study))
Web of Science	TS = ((“persistent left superior vena cava” OR “left superior vena cava”) AND (“congenital heart disease”) AND (prevalence OR incidence OR study))

**Table 2:** Characteristics of the included studies, including the risk of bias assessment.

Author (Year)	Population	Type	Sample	Demographics	Risk of Bias
Ari et al. (2017) [12]	Turkey	Imaging (US)	2663	1431 Male, 1232 Female, Mean Age: 3 (4.1) years (0–16)	Low
Bjerregaard and Laursen (1980) [13]	Denmark	Mixed (Autopsy-Interventional)	3667	Age: 0–15 years	High
Clarke et al. (2017) [14]	USA	Surgical	85	41 Male, 44 Female, Mean Age: 4.2 years (2–22)	High
Eldin et al. (2013) [15]	Saudi Arabia	Imaging (US)	738	Age: 0–16 years	Low
Erdogan et al. (2007) [16]	Turkey	Surgical	152	61 Male, 88 Females, Age: 1–65 years	Low
Galindo et al. (2007) [17]	Spain	Imaging (US)	504	-	High
Giuliani-Poncini et al. (2014) [18]	Switzerland	Surgical	371	Age: 0–16 years	Low
Kawada et al. (2021) [19]	America	Interventional	191	134 Male, 57 Female, Mean Age: 41.5 (14.8) years	Low
Kula et al. (2011) [20]	Turkey	Imaging (US)	1205	Mean Age: 4.5 years (0–17)	Low
Nagasawa et al. (2018) [21]	Japan	Imaging (US)	1920	1463 Male, 1378 Female, Age	Low
Pandey et al. (2020) [22]	India	Imaging (CTA)	973	699 Male, 274 Female, Mean Age: 4 years (0–12)	Low
Peng et al. (2024) [23]	China	Imaging (US)	10,817	-	High
Perles et al. (2013) [24]	Israel	Imaging (US)	8140	-	High
Postema et al. (2008) [25]	The Netherlands	Imaging (US)	1825	Mean Age: 2.9 (4.3) years (0–18)	Low
Taweevisit and Thorner (2019) [26]	Thailand	Autopsy	119	66 Male, 53 Female	Low
Toprak et al. (2024) [27]	Turkey	Surgical	4000	Age: 0–16 years	Low

**Table 3:** Statistical meta-analysis based on subgroup analysis for the prevalence of the persistent left superior vena cava (PLSVC) in congenital heart disease (CHD).

Parameter	PLSVC in CHD (%)
Overall	6.36
Asia (k = 10)	5.99
Europe (k = 4)	5.98
America (k = 2)	11.03
*p-value*	*0.5904*
Imaging (k = 10)	5.51
Autopsy (k = 2)	8.43
Surgical (k = 4)	8.93
*p-value*	*0.3241*
Echocardiography (k = 8)	4.59
Computed Tomography (k = 2)	10.26
*p-value*	*0.0092*
Pediatric (k = 13)	5.92
Adults/Mixed (k = 3)	5.83
*p-value*	*0.9456*
Ventricular Septal Defect (k = 4)	3.73
Patent Ductus Arteriosus (k = 4)	1.22
Tetralogy of Fallot (k = 4)	5.23
Transposition of Great Arteries (k = 4)	3.39
*p-value*	*0.1838*

## Data Availability

The data presented in this study are available on request from the corresponding author.

## Supplementary Materials

The following supporting information can be downloaded at https://www.mdpi.com/article/10.3390/life16081281/s1, Figure S1: Leave-one-out analysis forest plot for the prevalence of persistent left superior vena cava in congenital heart diseases, Figure S2: Baujat plot for the pooled prevalence estimate of persistent left superior vena cava in congenital heart diseases, indicating studies influencing the pooled prevalence estimate and the heterogeneity, Figure S3: Subgroup analysis forest plot for the prevalence of persistent left superior vena cava in congenital heart diseases according to their type; PRISMA_2020_Checklist.
