# Supplementary material for: Prevalence of Persistent Left Superior Vena Cava in Patients with Congenital Heart Disease: A Systematic Review with Meta-Analysis
Source: Life (Basel). 2026 Aug 3;16(8):1281. doi: 10.3390/life16081281 (PMC13514220; doi:10.3390/life16081281)
Supplement: Supplementary file 1 [file life-16-01281-s001.zip › life-4462964-supplementary figures.pdf]

Supplementary Material

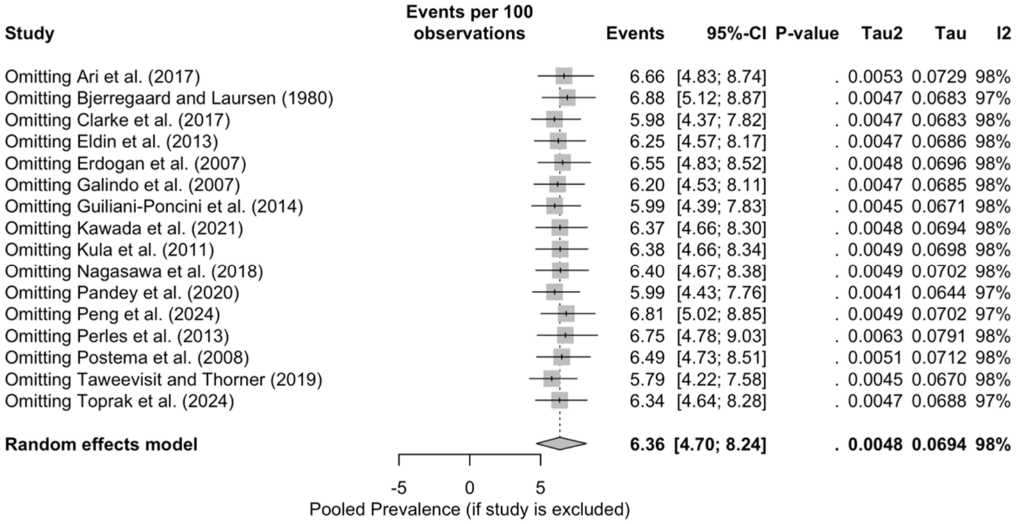

Supplementary Figure S1. Leave-one-out analysis forest plot for the prevalence of persistent left superior vena cava in congenital heart diseases.

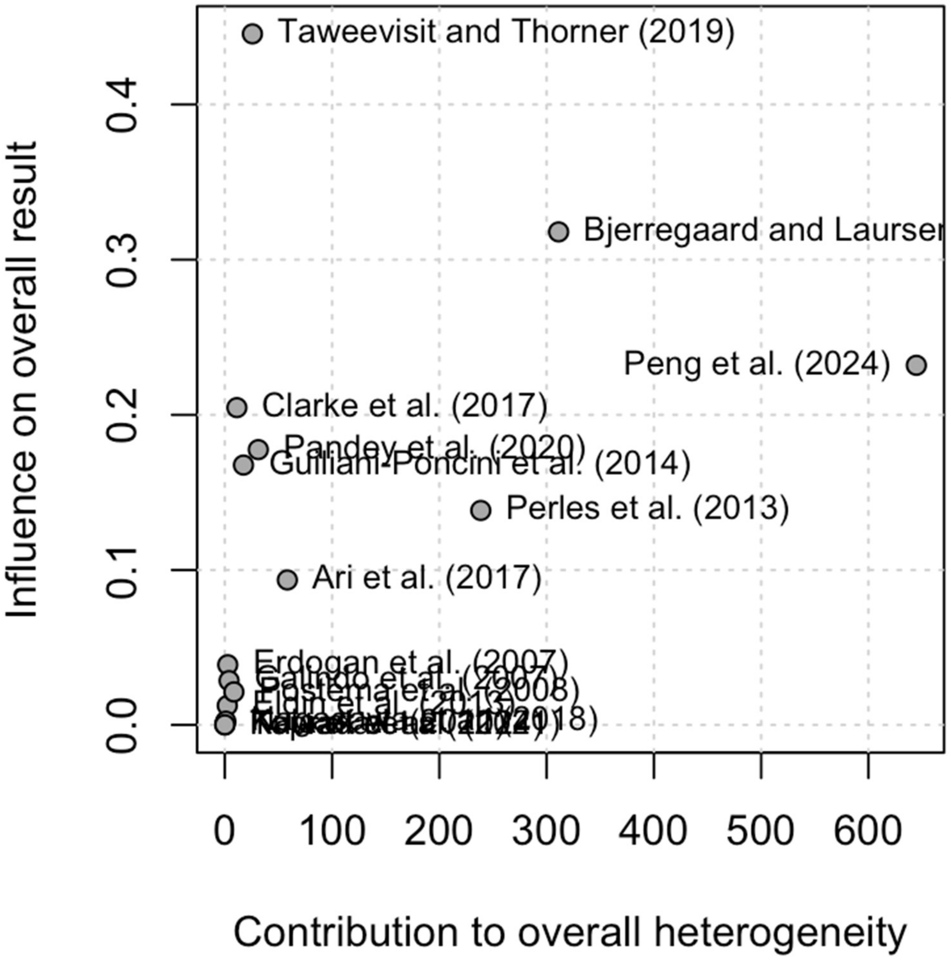

Supplementary Figure S2. Baujat plot for the pooled prevalence estimate of persistent left superior vena cava in congenital heart diseases, indicating studies influencing the pooled prevalence estimate and the heterogeneity.

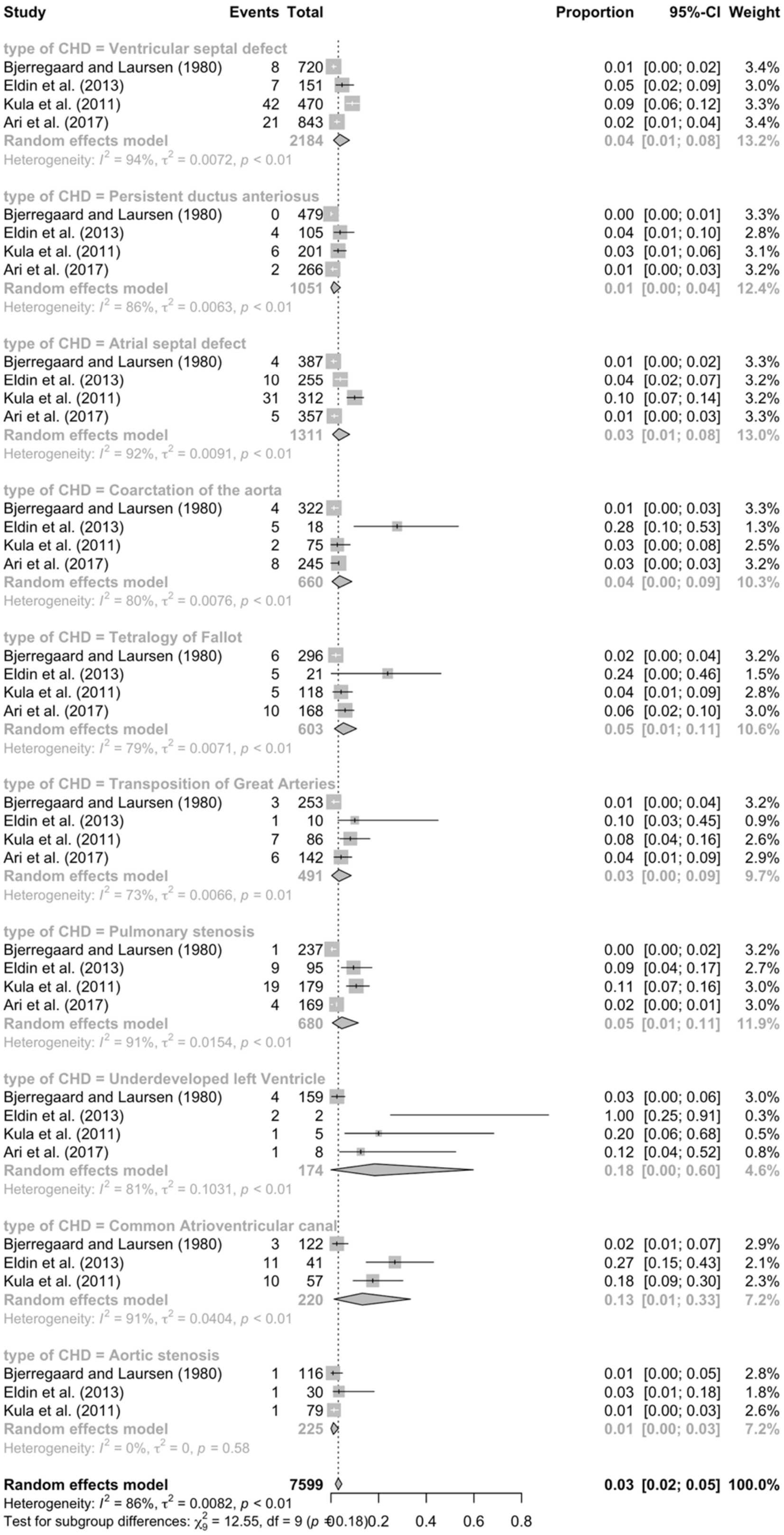

Supplementary Figure S3. Subgroup analysis forest plot for the prevalence of persistent left superior vena cava in congenital heart diseases according to their type.
